# Could African and Low- and Middle-Income Countries Contribute Scientifically to Global Cancer Care? The Answer Is Yes!

**DOI:** 10.1200/JGO.2016.003244

**Published:** 2016-03-09

**Authors:** Abdul Hannan

**Affiliations:** East Tennessee State University, Johnson City, TN

## To the Editor:

In his recent editorial in *Journal of Global Oncology*, Elzawawy^1^ has clearly enumerated important points in global cancer care, especially with regard to low- and middle-income countries (LMICs), where government policies, poverty, illiteracy, and lack of social justice are taking a toll not only on cancer care but also on other health care issues.

In my humble opinion, there is a need to develop more clinical trials tailored to the socioeconomic conditions of LMICs. For example, herceptin’s efficacy and survival advantage in treating breast cancer has been proven in well-developed prospective clinical trials.^2,3^ However, only a meager percentage of the population of LMICs can afford herceptin, which, according to world standards, must be taken for 1 year in an adjuvant setting. However, another study, from a Finnish group, suggests that only 9 weeks of herceptin use shows a disease-free and overall survival advantage (hazard ratios, 0.42 and 0.41).^4,5^ Although the study was not powered to illustrate the difference between the herceptin arms, it presents the possibility for a shorter duration of this costly treatment regimen, which almost 85% to 90% of patients with breast cancer in LMICs cannot afford. Obviously, the cost of 9 weeks of herceptin will be more affordable. Hopefully, the answer will come from the results of the ongoing Synergism or Long Duration (SOLD) study (NCT00593697). Conducting such trials in LMICs will help poor and needy patients and will reduce the cost of cancer care in already fragile health care systems.

As another example, imatinib (Gleevec, Novartis Pharmaceuticals, Basel, Switzerland) should be the first choice in newly diagnosed cases of CML, because it is time tested with >15 years of clinical experience, and it is also cheaper than new agents that put an additional cost on health care systems.

Finally, incorporating oncologists from LMICs in clinical trials will help them conduct their own trials according to their countries’ needs. Conducting clinical trials in low-income African and Asian countries will tap into 70% of potential trial patients, which will not only broaden the horizon of cancer care but also supply patients with cancer in developed countries with more robust data.

## Notes

**Abdul Hannan** No relationship to disclose

## References

[1] Elzawawy AM (2015). Could African and low– and middle–income countires contribute scientifically to global cancer care?. J Glob Oncol.

[2] Perez EA, Romond EH, Suman VJ Updated results of the combined analysis of NCCTG N9831 and NSABP B-31 adjuvant chemotherapy with/without trastuzumab in patients with HER2-positive breast cancer. J Clin Oncol.

[3] Goldhirsch A, Gelber RD, Piccart-Gebhart MJ (2013). 2 years versus 1 year of adjuvant trastuzumab for HER2-positive breast cancer (HERA): An open-label, randomised controlled trial. Lancet.

[4] Joensuu H, Kellokumpu-Lehtinen PL, Bono P (2006). Adjuvant docetaxel or vinorelbine with or without trastuzumab for breast cancer. N Engl J Med.

[5] Isik M, Dizdar O, Altundag K (2010). Two important determinants may play a role in the success of the FinHer Trial. J Clin Oncol.

